# Antibiotic Usage Pattern in Broiler Chicken Flocks in Germany

**DOI:** 10.3389/fvets.2021.673809

**Published:** 2021-06-07

**Authors:** Svetlana Kasabova, Maria Hartmann, Fritjof Freise, Katharina Hommerich, Stephani Fischer, Andreas Wilms-Schulze-Kump, Karl Rohn, Annemarie Käsbohrer, Lothar Kreienbrock

**Affiliations:** ^1^Department of Biometry, Epidemiology and Information Processing, WHO Collaborating Centre for Research and Training for Health at the Human-Animal-Environment Interface, University of Veterinary Medicine Hannover, Hannover, Germany; ^2^Veterinary Practice WEK, Visbek, Germany; ^3^Department Biological Safety, Federal Institute for Risk Assessment, Berlin, Germany; ^4^Unit of Veterinary Public Health and Epidemiology, Department for Farm Animals and Veterinary Public Health, University of Veterinary Medicine, Vienna, Austria

**Keywords:** antimicrobial use, weight at treatment, treatment frequency, broiler chicken flocks, daily dose

## Abstract

In this work, antimicrobial usage data from 2,546 commercial broiler chicken flocks originating from 37 farms are presented. Antimicrobial usage data at the flock level were based on mandatory documentation of antibiotic treatments in livestock in Germany, collected retrospectively for the time period of 2013–2018. The data encompasses all antimicrobial treatments during the fattening period of each flock, starting with the placement of day-old chicks at the barn. The aim of this analysis was to investigate antibiotic usage patterns in broiler chicken flocks in Germany, temporal trends in treatment frequency, the proportions of different antimicrobial classes and the weights of the broiler chickens at the time of treatment. The median treatment frequency over all flocks was six, and veterinary medicinal products belonging to nine different antimicrobial classes were used. Overall, the most frequently used classes were aminoglycosides (25.6%) and lincosamides (25.6%), followed by polypeptides (21.4%) and beta-lactams (16.2%). Over the 6 years evaluated, a considerable increase in the relative usage of lincosamides and aminoglycosides was observed. Compared to the first year of data collection, the percentage of treatments with fluoroquinolones, macrolides and polypeptides decreased in consecutive years. The median age of the broiler chickens at the time of treatment was 5 days, which corresponded to a median body weight at the time of treatment of 111 g, with substantial differences among various antimicrobial classes. We showed that in Germany, the median weight of broiler chickens at the time of treatment was substantially lower than the standard weight of broilers of 1,000 g proposed by the European Surveillance of Veterinary Antimicrobial Consumption. The median weight at treatment is very much influenced by the frequency of age-specific diseases. As different antimicrobial classes are used to combat these diseases, variations in the weight at treatment may have a considerable impact on the estimated treatment indicators. Additionally, a decrease in the relative usage of the highest-priority critically important antimicrobials, such as fluoroquinolones, macrolides and polypeptides, was shown, which might be the consequence of increasing awareness of the antibiotic resistance situation as well as of antibiotic monitoring and benchmarking systems currently running in Germany.

## Introduction

Poultry meat is a major food source for the rapidly growing global population (1). The European Union (EU) ranks third in the world's poultry meat production after the USA and Brazil. Within the EU, more than 70% of poultry meat is produced in six countries, namely, Poland, the UK, France, Germany, Spain and Italy (2). Under the conditions of intensive poultry production, the rational use of antimicrobial agents could play a vital role in the treatment of diseases (1, 3). However, there is a link between the development of antimicrobial resistance and the usage of antimicrobials (4), which leads to a growing public, political and scientific debate on the risks related to the usage of antimicrobials in the veterinary and agricultural sectors. There is no doubt that antimicrobial resistance is one of the top public health challenges of our century, and urgent measures need to be taken at all levels of society to reduce the impact and spread of antimicrobial resistance (5). In this context, better knowledge of antimicrobial treatments in livestock and robust monitoring and benchmarking systems are crucial for tackling antimicrobial resistance.

In Germany, antimicrobial usage (AMU) in broiler production is observed by a governmental antibiotic monitoring and benchmarking system. Since 2014, broiler holdings that keep an average of more than 10,000 animals have been required to submit detailed information about each antimicrobial treatment that the flock receives (6). Within this monitoring system, the treatment frequency (TF) is used as a benchmarking indicator, and the median and 75th percentile are defined as specific benchmarking thresholds. The TF per animal holding of the respective production type is calculated twice a year, and the benchmarking thresholds are published by the Federal Office of Costumer Protection and Food Safety. This system does not include any antimicrobial class-specific calculations or restrictions (7). The data obtained are detailed but are only allowed to be used for calculation of the TF, and no scientific evaluation of these data is permitted on a regular basis (8). Only recently a scientific evaluation for the purpose of assessing the effectiveness of the antimicrobial minimization concept was published (9).

Since 2012, the private company QS (QS Qualität und Sicherheit GmbH) has run an antibiotic monitoring system for its members. Within this approach, the treatment index in broiler chicken holdings is calculated per flock as a benchmarking indicator (10). A total of 1,932 broiler chicken holdings participated in the QS antibiotic monitoring system in 2019 (11).

Profound risk assessment of antimicrobial resistance development requires not only information about the amount of antimicrobials used in livestock, but also detailed knowledge of the treatment patterns in the course of the fattening cycle. This current study aims to investigate AMU patterns in 2,546 commercial broiler chicken flocks from 37 broiler chicken farms in Germany between 2013 and 2018. Our evaluation focuses on quantification of the AMU, the actual ages and weights of the animals at the time of treatment and the antimicrobial class profiles used over the duration of the growing period. To quantify AMU, we used the TF as a unit of measurement. We also describe temporal changes in AMU in broiler chicken flocks between 2013 and 2018, paying particular attention to the usage of the highest-priority critically important antimicrobials (HPCIAs).

## Materials and Methods

### Study Data

The VetCAb study started in 2008 as a feasibility project, aiming to investigate the practicality of implementing an antibiotic monitoring system in livestock in Germany (12). After a cross-sectional pilot project in 2011 (13), the study was continued as VetCAb-Sentinel using a longitudinal approach. The study population encompasses different livestock holdings. The type of study is an open cohort with ongoing recruitment of participants (14). AMU data are delivered by farmers and veterinarians to the VetCAb database and are related to mandatory documentation, application and delivery forms (ADFs), as legally required by the German Medicinal Products Act (14).

From the ongoing VetCAb-S project, data on AMU in 37 commercial broiler chicken farms supervised by one team of veterinarians who continuously participated in the study between 2013 and 2018 were included in this evaluation. AMU in those farms was evaluated at the flock level. Similar to Agunos et al., we defined the flock to be a homogeneous group of broiler chickens placed in a single production unit on the same day and raised and treated together as a group until preharvest sampling or slaughter (15). Therefore, one farm has at least one but more often several flocks at the same time. The allocation of the various flocks to the years evaluated was performed according to the date of placing the broiler chickens in the barn, regardless of the end of the fattening period.

For the time period of 2013 until 2018, AMU data from 2,546 broiler chicken flocks in total could be collected and analyzed. Data were collected retrospectively and encompassed all antimicrobial treatments during the complete fattening period of each flock, starting with placing of the day-old chicks at the barn. The ADF used to document antimicrobial treatments included detailed information about the number of animals treated, the initial treatment date, the application route, the name and amount of the antimicrobial drug applied, and the duration of the treatment.

Treatments with coccidiostats were excluded from this analysis. Antimicrobial therapies performed at the hatchery during the first 24–72 h of the chickens' life were also not included due to a lack of information.

### Overall Treatment Frequency

To quantify the frequency of antimicrobial treatments during the fattening period, we calculated the overall (TF) for every flock following Equation (1).

(1) $$TF=\frac{\sum_{treatments}\left(\#animals\;treated\;x\;\#treatment\;days\right)}{\#animals\;in\;the\;population}$$

A treatment is defined as the application of one antimicrobial compound to an animal. For this, veterinary medicinal products (VMPs) containing more than one antimicrobial class were considered separately for every antimicrobial class. The TF is an indicator of AMU in livestock at the farm level and indicates how many days an animal in the observed population is treated on average, e.g., how many used daily doses (UDDs) on average were administered to one animal within a given time period (16). This calculation method considers the actual number of animals treated (#animals treated) and the treatment duration (#treatment days) in the numerator and the estimate of the number of animals in the population under risk (#animals in the population) in the denominator. In Germany, information about the number of animals treated and the treatment duration is recorded in the ADF. The number of chickens stabled on day one was used to quantify the population at risk. Considering that in broiler production, treatments are usually performed on all animals present in the flock at the time of treatment, the number of animals treated is equal to the number of animals in the population in most cases. The number of animals stabled was not adjusted for losses due to mortality or preharvesting during the fattening period.

To calculate the temporal influence of the year on antibiotic consumption, we used a two-part random effects model (17). The first part modeled the probability of observing a TF > 0 by a logistic random intercept model. In the second part, given that TF is >0, a linear random intercept model was assumed for log TF. In both parts, the effect of the single farm was assumed to be random. The random effects from the first and second parts were modeled to be correlated. To fit the model, maximum likelihood estimation was used. The significance level was chosen to be *p* < 0.05. For the pairwise comparisons, the Bonferroni adjustment for multiple comparisons was used.

### Treatment Frequency per Antimicrobial Class

The relative TF per antimicrobial class was calculated considering all antimicrobial classes licensed for poultry in Germany, using a method introduced by Sjölund et al. and Schaekel et al. (18, 19). For this, the percentage of every antimicrobial class of the total TF per flock was calculated following Equation (2). To identify shifts in usage patterns over time, the relative TF per antimicrobial class was also calculated per year.

(2) $$\begin{array}{l} {\%TF}_{antimicrobial\;class}=\;\frac{\sum_{flocks}TF_{antimicrobial\;class}}{\sum_{flocks}\;TF}\;x\;100 \end{array}$$

The relative TF per antimicrobial class is only of limited informative value if the overall TF changes over time. To identify significant changes in the TF of the single antimicrobial classes, we used a binary logistic mixed model. The effect of a single farm was assumed to be random. The model was fitted using maximum likelihood estimation. The significance level was chosen to be *p* < 0.05. A Bonferroni adjustment for multiple comparisons was used for the pairwise comparisons.

### Treatment Patterns by Age and Estimated Body Weight

To describe the usage of antimicrobials in the course of the fattening period, we calculated the overall TF as well as the relative TF for each antimicrobial class used per week. For this, we divided the fattening period into 6 weeks. For every flock, we recorded the date of placing the 1-day-old chickens at the barn, assigning this date as the first day of the fattening period. To identify the age of the broiler at the time of treatment, we matched this information with the date of treatment start recorded in the ADF. Weight estimates are based on standard weight tables for the common breed (Ross 308) raised in our collective for as-hatched broilers (males and females combined) published in the Broiler Performance Objectives (20).

Statistical measures for the age and weight at the time of treatment were calculated per year under study, as well as over the complete study period. Estimates were also calculated for each antimicrobial class used to identify differences in the usage of the various antimicrobial classes over the duration of the fattening period.

All statistical evaluations mentioned above were performed with SAS®, version 9.4 TS level 1 M5 (SAS Institute Inc., Cary, NC, United States). The NLMIXED procedure with the adaptive Gaussian quadrature option was used to fit the two-part model. The binary logistic mixed model was fitted using the GLIMMIX procedure.

## Results

### Study Data

The total number of flocks included in this study over the observational period (2013–2018) was 2,546 and ranged from 362 to 473 per year. All flocks have been raised conventionally; the length of the production cycle was 42 days. Overall, 3,274 antimicrobial treatments were performed in the study population over the entire study period. Of these, 1,114 treatments consisted of VMP containing two antimicrobial classes (aminoglycosides/lincosamides or sulfonamides/trimethoprim). At the level of the antimicrobial class, 4,388 records were evaluated.

The number of day-old chickens placed per flock ranged from 9,000 to 66,000, and the total number of broiler chickens surveyed over the 6-year period was 78 M (78,010,902), ranging from 10.5 M (10,474,400) to 14.2 M (14,244,494) per year.

### Overall Treatment Frequency

The median TF over all 2,546 chicken flocks from 37 farms was six and ranged between four and six for the individual years. In total, 31.2% of all broiler chicken flocks did not use any antimicrobials at all (Table 1). At the farm level, no single farm was identified as having no antibiotic usage at all. The median treatment duration over the complete study period was 3 days, ranging from 1 to 5 days.

**Table 1:** Major measures of the statistical distribution of the overall TF at the flock level and percentage of flocks raised without AMU per year.

**Year**	**Number of flocks**	**Min**	**25%-Quantile**	**Median**	**75%-Quantile**	**Max**	**Number of flocks raised without antibiotics**	**Percentage of flocks raised without antibiotics**
2013	408	–	–	4.0	8.0	22.0	150	36.8
2014	362	–	–	5.8	6.0	21.3	117	32.3
2015	458	–	–	4.0	6.0	15.8	167	36.5
2016	473	–	–	6.0	6.0	16.4	144	30.4
2017	425	–	1.5	6.0	6.0	14.8	106	24.9
2018	420	–	–	6.0	6.0	19.5	110	26.2
Total	2,546	–	–	6.0	6.0	22.0	794	31.2

–*, observed zero; 0, zero by rounding*.

**Figure 1:**
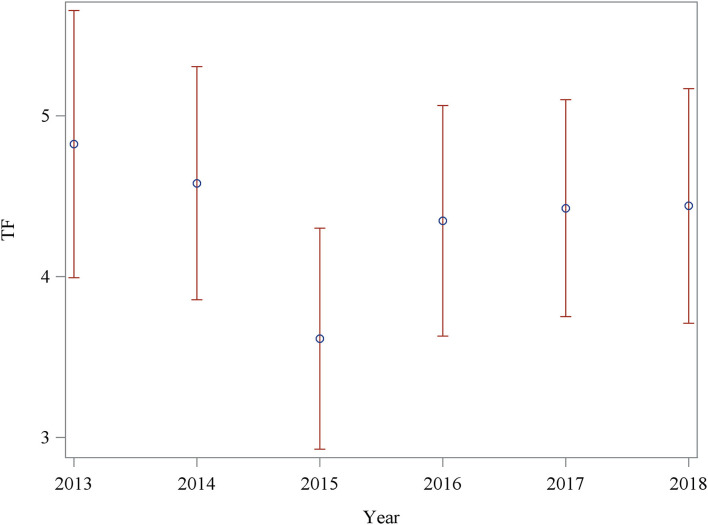
Estimated means of the TF over the years 2013–2018 with 95% confidence intervals.

Between 2014 and 2015, the mean of the TF significantly decreased, followed by an increase in 2016. Between 2013 and 2014, as well as within the subsequent years 2017 and 2018, no significant changes could be identified (Figure 1 and Table 2).

**Table 2:** Pairwise comparison of the mean TF between 2013 and 2018, Bonferroni adjusted.

**Comparison**	**Difference estimate**	**StdErr**	***t***	***p***	**Confidence bound**		***p*adj**
					**Lower**	**Upper**	
2014 vs. 2013	−0.2187	0.2831	−0.77	0.4449	−0.7934	0.3559	1.0000
2015 vs.2014	−0.9589	0.2464	−3.89	0.0004	−1.4591	−0.4588	0.0021
2016 vs. 2015	0.7078	0.2311	3.06	0.0042	0.2387	1.1769	0.0210
2017 vs. 2016	0.0750	0.2430	0.31	0.7593	−0.4182	0.5683	1.0000
2018 vs. 2017	0.0017	0.2541	0.01	0.9948	−0.5142	0.5175	1.0000

### Treatment Frequency per Antimicrobial Class

In total, 26 different VMPs belonging to nine antimicrobial classes were used. All treatments were administered via drinking water. No VMPs containing cephalosporins or pleuromutilins were reported. In our dataset, the usage of aminoglycosides (25.6%) and lincosamides (25.6%) had the highest relative TF, followed by polypeptides (21.4%) and beta-lactams (16.2%) (Table 3). The relative TF per antimicrobial class varied over time. The percentage of aminoglycosides and lincosamides increased significantly between 2013 and 2014 and then remained almost stable until 2018. Polypeptide usage dropped significantly in 2014 from 28.2% (2013) to 21.5% (2014) and remained at this level until 2016. In 2017, a slight increase was seen, with a consecutive drop in 2018 (Table 4). The relative percentage of macrolides decreased between 2013 and 2014 from 5.8 to 0.8% and remained under 1% over the study period. The relative usage of fluoroquinolones dropped significantly in 2014 from 8.5 to 2.4%. The relative use of tetracyclines was very low over the complete observation period. The relative use of HPCIA, i.e., fluoroquinolones, macrolides and polypeptides (21) [VMPcontaining cephalosporins (3rd-generation and higher) or glycopeptides are not labeled for use in poultry in Germany; vetidata.de (22)], accounted for 26.2% of the overall TF over the complete study period. In 2013, 42.5% of the TF was represented by HPCIA, and in 2018, the proportion was only 19.6% (Table 3).

**Table 3:** Relative (TF%) and absolute (total in all flocks) treatment frequency (STF) per antimicrobial class and year.

**Antimicrobial class**	**2013**		**2014**		**2015**		**2016**		**2017**		**2018**		**Overall 2013–2018**
	**TF%**	**STF**	**TF%**	**STF**	**TF%**	**STF**	**TF%**	**STF**	**TF%**	**STF**	**TF%**	**STF**	**TF%**
Aminoglycosides	16.0	321.5	26.2	431.4	25.6	444.9	29.4	623.5	29.8	629.2	26.0	553.0	25.6
Beta-Lactams	18.2	365.7	15.7	258.5	20.6	358.0	16.7	354.2	13.2	278.7	13.8	293.5	16.2
Fluoroquinolones^*^	8.5	170.8	2.4	39.5	2.8	48.7	2.3	48.8	3.1	65.5	1.5	31.9	3.4
Lincosamides	16.0	321.5	26.2	431.4	25.6	444.9	29.4	623.5	29.8	629.2	26.4	561.6	25.6
Macrolides^*^	5.8	116.6	0.8	13.2	0.6	10.4	0.4	8.5	0.9	19.0	0.0	0	1.4
Polypeptides^*^	28.2	566.7	21.5	354.0	24.0	417.1	18.0	381.7	19.3	407.5	18.1	385.0	21.4
Sulfonamides	3.7	74.4	3.6	59.3	0.3	5.2	1.9	40.3	1.7	35.9	6.4	136.1	3.0
Tetracyclines							0.1	2.1	0.4	8.4	1.3	27.7	0.3
Trimethoprim	3.7	74.4	3.6	59.3	0.3	5.2	1.9	40.3	1.7	35.9	6.4	136.1	3.0
Total	100	2,009.6	100	1,646.7	100	1,738.0	100	2,120.7	100	2,111.5	100	2,127.1	100
Total HPCIA^*^	42.5	854.1	24.7	406.7	27.4	476.2	20.7	439.0	23.3	492	19.6	416.9	26.2

* *HPCIA due to WHO-AGISAR definition, i.e., fluoroquinolones, macrolides and polypeptides [VMP containing cephalosporins (3rd-generation and higher) or glycopeptides are not labeled for use in poultry in Germany (21, 22)]*.

**Table 4:** Estimated proportion of the AMU per antimicrobial class; estimated least square means with confidence interval; pairwise comparison of the usage between consecutive years.

**Antimicrobial class**	**2013**	**2014**	**2015**	**2016**	**2017**	**2018**
Aminoglycosides	0.228 (0.157–0.320)	0.409 (0.301–0.525)	0.289 (0.203–0.393)	0.403 (0.300–0.516)	0.430 (0.323–0.544)	0.349 (0.253–0.459)
	*p* < 0.001 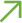		*p* = 0.024 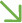	*p* = 0.018 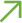	*p* = 1.000	*p* = 0.511
Beta-Lactams	0.278 (0.211–0.357)	0.231 (0.168–0.308)	0.288 (0.219–0.370)	0.249 (0.186–0.325)	0.266 (0.199–0.345)	0.211 (0.1550–0.282)
	*p* = 1.000		*p* = 1.000	*p* = 1.000	*p* = 1.000	*p* = 1.000
Fluoroquinolones	0.842 (0.043–0.158)	0.020 (0.008–0.048)	0.022 (0.010–0.049)	0.018 (0.008–0.041)	0.022 (0.010–0.050)	0.011 (0.004–0.026)
	*p* = 0.005 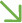		*p* = 1.000	*p* = 1.000	*p* = 1.000	*p* = 0.747
Lincosamides	0.227 (0.156–0.319)	0.408 (0.301–0.524)	0.288 (0.203–0.392)	0.402 (0.299–0.515)	0.429 (0.322–0.544)	0.355 (0.258–0.465)
	*p* < 0.001 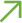		*p* = 0.024 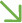	*p* = 0.017 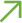	*p* = 1.000	*p* = 0.759
Macrolides	0.035 (0.014–0.086)	0.007 (0.002–0.022)	0.004 (0.001–0.014)	0.003 (0.001–0.013)	0.019 (0.007–0.047)	–
	*p* = 0.005 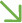		*p* = 1.000	*p* = 0.762	*p* = 0.087	–
Polypeptides	0.413 (0.331–0.500)	0.285 (0.215–0.369)	0.309 (0.238–0.391)	0.251 (0.189–0.325)	0.349 (0.272–0.435)	0.252 (0.189–0.328)
	*p* = 0.006 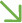		*p* = 1.000	*p* = 0.814	*p* = 0.031 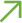	*p* = 0.048 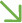
Sulfonamides	0.0459 (0.028–0.075)	0.037 (0.021–0.065)	0.003 (0.001–0.015)	0.031 (0.018–0.053)	0.035 (0.020–0.060)	0.106 (0.073–0.152)
	*p* = 1.000		*p* = 0.021 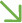	*p* = 0.0491 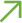	*p* = 1.000	*p* = 0.001 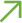
Trimethoprim	0.046 (0.028–0.075)	0.037 (0.021–0.065)	0.003 (0.001–0.015)	0.031 (0.018–0.053)	0.035 (0.020–0.060)	0.106 (0.073–0.152)
	*p* = 1.000		*p* = 0–021 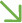	*p* = 0.049 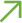	*p* = 1.000	*p* = 0.001 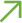

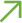
*–significant increase*, 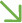
*–significant decrease*.

### Treatment Patterns by Age and Estimated Body Weight

To gain deeper insight into the treatment of broiler chickens during the production cycle, data were stratified by the age at treatment. The median age of the broiler chickens at the time of treatment was 5 days over the complete study period and varied between 2 and 6 days depending on the observation year, with an outlier of 19 days in 2013 (Table 5).

**Table 5:** Major measures of the statistical distribution of the age of the broiler chickens in days at the time of treatment start per year.

**Year**	**Number of records**	**Min**	**25% - quantile**	**Median**	**75% - quantile**	**Max**
2013	619	1	2	19	25	38
2014	446	1	1	6	25	39
2015	503	1	1	2	22	38
2016	586	1	1	5	24	38
2017	602	1	1	5	24	39
2018	518	1	1	2	17	40
Total	3,274	1	1	5	24	40

**Figure 2:**
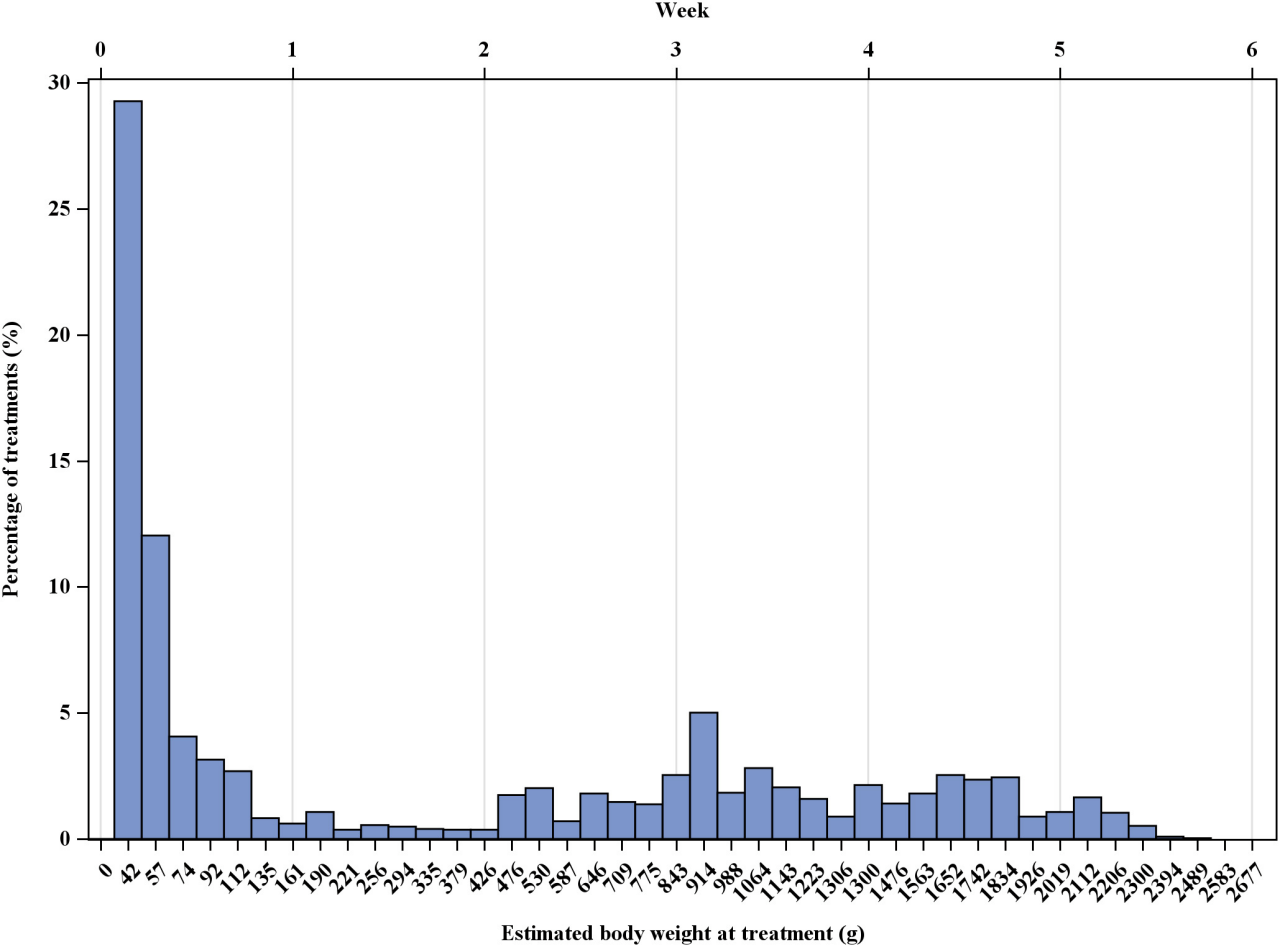
Statistical distribution of the treatments by the estimated body weight of the broiler chickens in g over the entire study period (2013–2018).

The entire distribution of treatments by body weight of the broiler chicken is displayed in Figure 2. It is a right-skewed distribution with more than 50% of treatments within the first week of fattening. From the age of the animals at the time of treatment, the estimated body weight of the animals was derived. The median body weight at treatment was 111 g over the entire study, ranging from 57 to 719 g over the years (details in Supplementary Table 1).

Upon stratifying these distributions by antimicrobial class, different patterns occurred. The median age at the start of treatment varied for the individual antimicrobial classes from 1 day for aminoglycosides and lincosamides to 22 days for beta-lactams and macrolides (Table 6). The age at the time of treatment per antimicrobial class and year is displayed in Supplementary Table 2. The median body weight of the broiler chickens at the time of treatment also varied between 42 and 914 g depending on the antimicrobial class used (Supplementary Table 3). The median body weight at the time of treatment per antimicrobial class and year is displayed in Supplementary Table 4.

**Table 6:** Major measures of the statistical distribution of the age of the broiler chickens in days at the time of treatment per antimicrobial class.

**Antimicrobial class**	**Number of records**	**Min**	**25% - Quantile**	**Median**	**75% - Quantile**	**Max**
Aminoglycosides	982	1	1	1	2	24
Beta-Lactams	845	1	7	22	28	39
Fluoroquinolones	136	1	2	3	6	38
Lincosamides	984	1	1	1	2	24
Macrolides	76	1	20	22	28	38
Polypeptides	1,087	1	5	21	29	40
Sulfonamides	132	1	1	4	11	36
Tetracyclines	14	1	1	3	5	26
Trimethoprim	132	1	1	4	11	36

As displayed in Table 6, the antibiotic class applied varied substantially with the age of the broiler chickens. Therefore, the relative TF per antimicrobial class was additionally stratified by fattening week (Table 7). The median overall TF per week decreased steadily during the fattening period, starting at 3.72 in the first week of broiler chicken life. From the second week to the end of the fattening period, the median TF remained constant at the value of zero. The mean of the overall TF varied over the fattening period between 0.07 and 3.32 and reached its highest level in the first and fourth weeks of the fattening period (Table 7).

**Table 7:** Relative TF per antimicrobial class and fattening week.

**Antibiotic class**	**Week 1**	**Week 2**	**Week 3**	**Week 4**	**Week 5**	**Week 6**
	**Median TF = 3.72**	**Median TF = 0**	**Median TF = 0**	**Median TF = 0**	**Median TF = 0**	**Median TF = 0**
	**Mean TF = 3.32**	**Mean TF = 0.13**	**Mean TF = 0.36**	**Mean TF = 0.46**	**Mean TF = 0.29**	**Mean TF = 0.07**
	**%**	**%**	**%**	**%**	**%**	**%**
Aminoglycosides	35.2	6.8	0.0	0.6	0.0	0.0
Beta-Lactams	7.3	26.0	44.4	41.9	34.8	40.9
Fluoroquinolones	4.2	10.6	0.4	0.5	1.3	0.3
Lincosamides	35.3	6.8	0.0	0.6	0.0	0.0
Macrolides	0.5	0.0	2.7	6.1	3.4	2.2
Polypeptides	11.1	32.1	47.4	47.1	53.5	53.1
Sulfonamides	3.0	8.9	2.5	1.4	3.5	1.7
Tetracyclines	0.4	0.0	0.0	0.5	0.0	0.0
Trimethoprim	3.0	8.9	2.5	1.4	3.5	1.7
All	100.0	100.0	100.0	100.0	100.0	100.0

There was also a shift in the percentages of the different antimicrobial classes used during the fattening period. In the first fattening week the combination of aminoglycosides and lincosamides (35.2 and 35.3%) had the highest TF, followed by polypeptides (11.1%) and beta-lactams (7.3%). Starting from the second week of fattening, the usage of polypeptides had the highest TF, followed by the usage of beta-lactams. A peak in the relative fluoroquinolone, sulfonamide and trimethoprim usage was seen in the second fattening week, when these antimicrobial classes accounted for 10.6 and 8.9%, respectively, of the overall TF. The usage of macrolides reached its peak of 6.1% in the fourth fattening week. The relative TF for tetracyclines remained at a very low level during the entire fattening period (Table 7).

**Figure 3:**
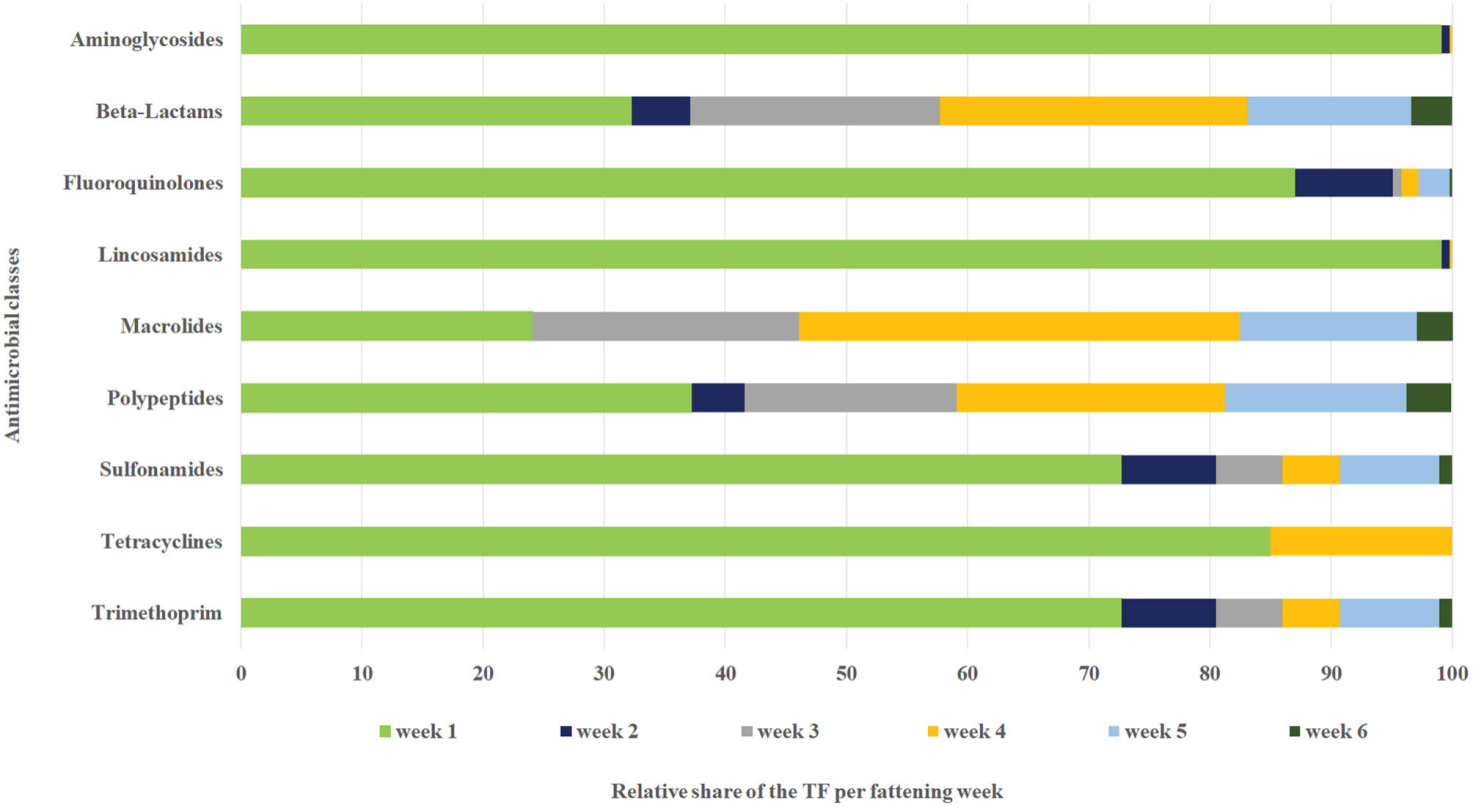
Relative TF per antimicrobial class and fattening week.

As displayed in Figure 3, aminoglycosides, lincosamides and fluoroquinolones were applied in the highest percentages in the first fattening week, and beta-lactams, macrolides and polypeptides were given over the complete fattening period.

## Discussion

### Study Design

In the present work, data on AMU from 2,546 conventional broiler chicken flocks raised in 37 panel farms between 2013 and 2018 and supervised by one team of veterinarians were evaluated, aiming to describe detailed AMU patterns in German broiler production. Over the 6-year study period, a total of 78 million broiler chickens was surveyed, which represents ~2.1% of the total number of broiler chickens slaughtered in Germany between 2013 and 2018 (23).

The study population enrolled has to be considered a convenience sample due to their voluntary participation. Hence, our dataset is prone to selection bias, although the farms under observation represent a typical production environment in northwestern Germany. This is, for example, demonstrated by the usual production numbers of the entire German broiler production, in which the average production cycle is 42 days for conventionally fattened broilers (42 days in our sample) (24). According to data from the German Federal Statistical Office, there were 3,330 broiler farms in Germany in 2016, with 93,791,251 broiler chickens overall. On average, 28,165 broiler chickens per farm were kept nationwide, which is consistent with the average number of stabled animals in our study cohort (between 29,330 and 33,516) (25).

The records used are mandatory for the documentation of antimicrobial use in Germany. The format is binding and includes detailed information on the number of animals treated, duration of treatment and amount and name of the drugs applied. Thus, we expect information bias due to insufficient documentation to not play a major role in the outcome.

### Overall Treatment Frequency

The median TF in our study population was six, i.e., during the fattening period, each broiler received six UDD on average. We also found an increase in TF in 2016 compared to previous years.

To the best of our knowledge, in Germany, there are no antimicrobial treatments applied at the hatchery, so we did not consider any underestimations of the TF due to underreporting of antimicrobial treatments at the hatchery.

To calculate the TF, the denominator was stated as the number of day-old chickens placed in the barn on day one of the fattening period. This was not adjusted for losses due to mortality during the fattening period, which, in theory, causes an underestimation of the measure. However, considering an average mortality rate of 3% during the fattening period (24), which is ~0.1% per day, this causes an average bias of 0.2 in the TF, which we consider to be negligible.

Furthermore, treatments performed after the preharvest sampling day do not encompass the entire broiler population stabled at day one of the fattening period. In Germany, broilers are often reared to the maximum stock density permitted and thinned afterward. Approximately 25% of the flock is removed for slaughter at approximately day 31 to lower the density and to allow the remaining birds to grow without exceeding the maximal stock density of 39 kg/m^2^ (24). Due to the calculation procedure used in these cases, systematic underestimation of the TF is possible as well. This takes into account flocks with preharvesting in which treatments were performed after day 31. In our dataset, these constitute ~10% of all treatments. In this 10%, there is an overestimation of the population under risk of ~25%. Referring to the complete dataset, the denominator is relatively biased to a maximum of 6.25%, which leads to some underestimation of the TF.

In a study in Belgian broiler flocks, the authors found that a broiler received approximately one UDD on average for 5 days of the growing period (26). In our calculation method, VMP containing two or more different antimicrobial substances in a fixed combination (namely, lincomycin/spectinomycin and sulfonamide/trimethoprim) were entered in the calculation as a value of two, producing a 2-fold higher number of UDDs in those cases where fixed combinations were used. In our dataset, 34% of all VMPs used are combinations that mainly explain the difference in both outcomes.

The same applies when comparing our results to the outcome of the private QS antibiotic monitoring system, where the median therapy index is calculated semiannually. Between the second half of 2014 and the second half of 2018, the therapy index for broiler flocks recorded by QS was as follows: 4.41, 3.81, 2.57, 2.71, 2.94, 3.47, 3.77, 3.99, and 4.30 (27). In this system, fixed combinations of lincomycin/spectinomycin and sulfonamide/trimethoprim are handled as one when calculating the number of UDDs, which results in a lower number of UDDs compared to our calculation.

Within the German governmental antibiotic monitoring system, the TF is calculated per farm twice a year. Our data are generally structured per flock; however, within our model calculations, the farm structure was considered a random variable. Taking this into account, the same trend can be observed concerning the increasing TF. Here, after a short-term drop between the second half of 2014 and the first half of 2015, the TF steadily increased. Within our dataset, there was a significant drop in TF between 2013 and 2015, with the lowest values in 2015, and in 2016, there was an increase in TF.

A total of 31.5% of the 3,274 flocks evaluated did not receive any antimicrobials, showing that under the conditions of conventional broiler chicken production in Germany, it is possible to raise antibiotic-free flocks. According to non-published data from the German poultry sector, 30% of the broiler chicken flocks raised in Germany did not receive any antibiotics (personal communication QS), which is a quite similar result.

According to the official monitoring system in Germany, 6.3% of the broiler holdings managed to raise all flocks in the observation period from July 2014 to December 2017 without any AMU. For the individual half-year period, the proportion of broiler holdings without recorded antimicrobial use ranged between 17.2 and 23.6% (9). However, for our data, within the entire study period, no single farm was identified as having no antibiotic use.

Persoons et al. found 25% of the monitored production cycles to be able to grow broilers without any antimicrobials. A Moroccan study on the consumption of antimicrobials in broiler production reported that 93% of the observed flocks received at least one antimicrobial treatment during the fattening period (28). The authors considered this high level of AMU to be due to poor rearing practices, low-quality day-old chicks and feed and a lack of efficient official oversight. A study performed by Joosten et al. aiming to quantify AMU in broiler chicken flocks in 9 European countries found 37% of 181 observed flocks to have not used antimicrobials at all. The percentage of flocks without AMU varied between 5 and 85% depending on the country (29).

### Treatment Frequency per Antimicrobial Class

Overall, 26 VMPs licensed for poultry were used in the study cohort. No off-label use could be detected over the observation period. In 34% of all treatments, fixed combinations were applied.

We found that aminoglycosides and lincosamides used in a combination product, polypeptides and beta-lactams represented the highest relative TFs. Within the scientific evaluation of the Germany-wide antibiotic usage data, it could also be shown that antibiotic usage in broilers in Germany is dominated by the use of a combination of aminoglycosides and lincosamides, beta-lactams, and polypeptides (30).

In the evaluation report, a significant increase in the usage of the combination of aminoglycosides and lincosamides in 2017 compared to 2015 could also be observed, as well as a significant decrease in the usage of the combination of sulfonamides and trimethoprim, macrolides, beta-lactams, polypeptides and tetracyclines. In contrast to our study, no significant trend in the usage of fluoroquinolones was observed (30). However, it must be considered that a longer observation period is covered in our study (2013–2018). Within our study, a significant drop in the relative TF of fluoroquinolones was seen between 2013 and 2014. In the time period between 2015 and 2019, the relative usage of fluoroquinolones in our cohort also remained stable. Taking this into account, our study supports the interpretation that the new regulations put in place in 2014 had initial temporary effects on antimicrobial use in broilers, but the recent situation in Germany seems to be stable.

A Canadian study on AMU in broilers investigated the number of defined daily doses (DDD) per 1,000 chicken days at risk and found the top three antimicrobial classes used to be bacitracin, followed by streptogramin and trimethoprim-sulfonamides (15). In this study, Agunos et al. developed Canadian DDDvet standards in mg_drug_/kg_animal_ using the 1 kg standard body weight for broiler chickens at treatment proposed by European Surveillance of Veterinary Antimicrobial Consumption (ESVAC). Comparing these results with ours, it needs to be kept in mind that there could be systematic differences between the UDD and the DDD, which may limit the comparability of the data (31). The same applies when comparing our results with Caucci et al., in which AMU in broilers was measured in DDDvet/PCU. Caucci et al. found the highest number of DDDvet/PCU in 2015 to be allocated to the usage of beta-lactams, polymyxins and sulfonamides (32). Joosten et al. reported polymyxins (26%), extended-spectrum aminopenicillins (26%) and fluoroquinolones (18%) to be the most commonly used antimicrobials in their collective (29).

Three out of five antimicrobial classes ranked by the World Health Organization (WHO) as HPCIA were used in our study (21). There are no VMP containing cephalosporins (3rd generation and higher) or glycopeptides labeled for use in poultry in Germany [www.vetidata.de (22)], and no usage was recorded in our dataset. During the study period, 26.2% of the treatments involved the use of HPCIA, namely, fluoroquinolones (3.4%), macrolides (1.4%) and polypeptides (21.4%), whereby a considerable reduction in the usage of these antimicrobial classes was observed between 2013 and 2018, i.e., fluoroquinolones (−7%), macrolides (−5.8%) and polypeptides (−10.1%). Within the report of the Federal Ministry of Food and Agriculture on the evaluation of the Antibiotics Minimization Concept introduced with the 16th Act to Amend the Medicinal Products Act (16th AMG Amendment), a report of the federal states on the findings and experiences of the competent authorities regarding the implementation of the 16th AMG Amendment and a nationwide survey on the experiences of animal keepers and veterinarians with the provisions and measures of the 16th AMG Amendment was integrated. Here, growing awareness among animal keepers and veterinarians when using antibiotics in recent years is confirmed. Within the nationwide survey, almost 90% of the veterinarians stated that there was increased awareness about the use of antibiotics among animal keepers. More than 60% agreed that legal regulation had helped reduce the use of antibiotics. The official authorities of the federal states also found an increase in preventive measures to avoid infections (e.g., vaccinations) and to optimize management and animal health (9). Although no details per farm and flock are reported for this in our study, we assume a general growing awareness of the global antimicrobial resistance situation among all actors involved in poultry production and marketing, as a consequence of the currently running antibiotic monitoring and benchmarking system in Germany.

We further showed that the percentages of the different antimicrobial classes used changed during the fattening period. In the first week of fattening, treatments were dominated by the usage of aminoglycosides and lincosamides, followed by the usage of polypeptides. Detailed treatment data for broiler chickens in the course of the fattening period are rare. Agunos et al. found lincomycin-spectinomycin to be used in 26% of the flocks *per injectionem* at the hatchery to target avian pathogenic *E. coli* (APEC) and enteric diseases (15). In our study, lincomycin-spectinomycin treatments were performed via drinking water on the first day (median value) of the fattening period (the weight at the time of treatment was 42 g). Similar results were reported by the EFFORT consortium. Here, lincomycin-spectinomycin was only used in treatments initiated on days 1 and 2 of the fattening period. In contrast, extended-spectrum penicillin treatments were registered on 29 different days during the production period (29).

### Treatment Patterns by Age and Estimated Body Weight

The median age of the broiler chickens at the time of treatment was 5 days. This corresponds to a median weight at the time of treatment of 111 g. The daily gain and body weight were derived from the Ross 308 broiler performance objectives for as hatched broiler chickens, which is the most common breed used in our study. These objectives indicate for performance achievable under good management and environmental conditions (20). Suboptimal husbandry conditions at the farm or disease outbreaks can result in a lower performance and could have led to overestimation of the weight at the time of treatment in our results. Furthermore, there are uncertainties in the age estimation due to the duration of the hatch window. The hatch window of broiler chickens ideally lasts 24 h, during which no more than 25% of the broiler chickens should hatch 23 h before pull and 75% of chicks should hatch ~13 h before pull (33). Therefore, up to 25% of the flock's age will be underestimated by 24 h. Furthermore, we did not take the treatment duration into account, and the weight at the time of treatment was the estimated weight on the first day of antibiotic treatment. The median treatment duration in our dataset was 3 days. Therefore, an underestimation of the weight on the following 2 days of treatment is possible. However, overall, these biases may be considered to be negligible for the interpretation of the results.

According to non-published data from the poultry sector (QS, personal communication) in Germany, over 50% of all treatments take place in the first 7 days of the fattening period, which corresponds with our results. Joosten et al. reported 49% of the overall AMU to be administered within the first week of fattening, considering AMU in broiler flocks raised in nine different EU countries (Belgium, Bulgaria, Denmark, France, Germany, Italy, Poland, Spain and the Netherlands) (29). The standard weight at treatment proposed by ESVAC for calculation of national indicators based on antimicrobial amounts for broiler chickens is 1 kg and substantially higher than the median weight observed in our study (34). The weight of 1 kg is also applied in the Dutch MARAN report (35). In a Belgian study, the weight of broiler chickens was estimated at 984 g and rounded to 1 kg (26).

In a previous work, we showed that discrepancies between the standard weight and the actual weight of the animals at the time of treatment could have a significant impact on the outcome of AMU quantification based on standard weights and can lead to under- or overestimation of AMU, depending on the standardization method used (31). A cross-country comparison of antimicrobial use based on DDD using a standard weight of 1 kg would lead to underestimation of the antimicrobial use in countries where treatment patterns in broiler chickens are dominated by treatments within the first fattening week, as is the case in our study collective.

## Conclusion

A sustained reduction in the overall antimicrobial TF in poultry farms seems to not yet have been achieved. After a short-term drop in AMU at the beginning of the study period, the TF returned to the initial values. However, a considerable reduction in the proportional use of the HPCIAs between 2013 and 2014 was observed. This might be the consequence of increasing awareness of the global antibiotic resistance situation as well as of actions due to expected restrictions in the context of the benchmarking system in Germany, which started in 2014. The reduction of HPCIAs seemed to have resulted to a compensatory rise in other classes.

The median weight of the broiler chickens at the time of treatment in our study was substantially lower than the standard weight for broilers of 1,000 g proposed by ESVAC. We consider the large deviations in age and weight at the time of treatment between different antimicrobial classes to have a significant impact on the calculated indicator and interpretation of results. Using UDDs overcomes this limitation, whereas assessment of AMU when standard weights are used and no information on the actual weight of the animals at the time of treatment is available might give a biased picture of antimicrobial use per flock.

## Data Availability Statement

The data were collected on an individual basis from farmers and veterinary practitioners. Each participant gave written consent with the understanding that data would not be transferred to a third party. Therefore, any data transfer to interested persons is not allowed without an additional formal contract. Data are available to qualified researchers who sign a contract with the University of Veterinary Medicine Hannover. This contract will include guarantees to the obligation to maintain data confidentiality in accordance with the provisions of the German data protection law. Currently, there exists no data access committee or another body who could be contacted for the data. But for this purpose, a committee will be founded. This future committee will consist of the authors as well as members of the University of Veterinary Medicine Hannover and members of the funding institution (Federal Institute for Risk Assessment). Interested cooperative partners, who are able to sign a contract as described above, may contact: LK, lothar.kreienbrock@tiho-hannover.de.

## Author Contributions

SK and LK: conceptualization, formal analysis, investigation, methodology, and writing—original draft. SK and MH: data curation and validation. LK: funding acquisition and supervision. SK and KH: project administration. MH, FF, and KR: software. SK, MH, KR, and FF: visualization. SK, FF, KH, SF, AW-S-K, AK, and LK: writing—review and editing. All authors contributed to the article and approved the submitted version.

## Conflict of Interest

The authors declare that the research was conducted in the absence of any commercial or financial relationships that could be construed as a potential conflict of interest.

## Abbreviations

ADF: Application and Delivery Form
AMU: Antimicrobial Usage
APEC: Avian Pathogenic *E. coli*
DCDvet: Defined Course Dose for animals
DDD: Defined Daily Dose
DDDvet: Defined Daily Dose for animals
ESVAC: European Surveillance of Veterinary Antimicrobial Consumption
EU: European Union
HPCIA: Highest Priority Critically Important Antimicrobials
QS: QS Qualität und Sicherheit GmbH
TF: Treatment Frequency
UDD: Used Daily Dose
VetCAb-S: Veterinary Consumption of Antibiotics Sentinel
VMP: Veterinary Medicinal Products
WHO: World Health Organization.
