# Supplementary material for: Antibiotic Usage Pattern in Broiler Chicken Flocks in Germany
Source: Front Vet Sci. 2021 Jun 7;8:673809. doi: 10.3389/fvets.2021.673809 (PMC8215671; doi:10.3389/fvets.2021.673809)
Supplement: Supplementary file 1 [file Data_Sheet_1.pdf]

Supplement 1:

Supplement 1, Table 1: Major measures of the statistical distribution of the estimated weight in kg at the start of treatment over the years

year	Body weight in kg at start of treatment							
	number of records	mean	min	IQR	25% quantile	median	75% quantile	max
**2013**	619	0.721	0.042	1.105	0.057	0.719	1.162	2.334
**2014**	446	0.611	0.042	1.120	0.042	0.134	1.162	2.429
**2015**	503	0.531	0.042	0.887	0.042	0.057	0.929	2.334
**2016**	586	0.616	0.042	1.040	0.042	0.111	1.082	2.334
**2017**	602	0.607	0.042	1.040	0.042	0.111	1.082	2.429
**2018**	518	0.432	0.042	0.551	0.042	0.057	0.593	2.524
**Total**	3,274	0.592	0.042	1.040	0.042	0.111	1.082	2.524

Supplement 1, Table 2: Major measures of the statistical distribution of the age of the broiler chickens in days at the time of treatment per antimicrobial class over time

Antimicrobial class	year	number of records	min	25% - quantile	median	75% - quantile	max
**Aminoglycosides**	**2013**	94	1	1	1	1	24
	**2014**	140	1	1	1	1	5
	**2015**	148	1	1	1	2	22
	**2016**	209	1	1	1	2	5
	**2017**	211	1	1	1	2	10
	**2018**	180	1	1	1	2	13
**Beta-Lactams**	**2013**	163	1	19	22	28	36
	**2014**	118	1	12	24	30	39
	**2015**	152	1	2	20	24	38
	**2016**	161	1	15	22	30	38
	**2017**	136	1	14	22	30	38
	**2018**	115	1	1	10	23	35
**Fluoroquinolones**	**2013**	52	1	1	3	5	33
	**2014**	14	1	2	4	7	8
	**2015**	19	1	1	4	26	38
	**2016**	17	1	2	3	4	34
	**2017**	22	1	3	4	4	9
	**2018**	12	1	2	6	17	33
**Lincosamides**	**2013**	94	1	1	1	1	24
	**2014**	140	1	1	1	1	5
	**2015**	148	1	1	1	2	22
	**2016**	209	1	1	1	2	5
	**2017**	211	1	1	1	2	10
	**2018**	182	1	1	2	2	13
**Macrolides**	**2013**	49	1	19	22	31	38
	**2014**	6	16	16	21	24	24
	**2015**	4	22	22	22	24	25
	**2016**	4	20	20	20	22	23

Antimicrobial class	year	number of records	min	25% - quantile	median	75% - quantile	max
	**2017**	13	18	24	24	30	33
**Polypeptides**	**2013**	235	1	9	21	28	38
	**2014**	149	1	7	22	29	37
	**2015**	178	1	2	19	25	38
	**2016**	177	1	12	22	30	38
	**2017**	199	1	8	22	30	39
	**2018**	149	1	2	14	29	40
**Sulfonamides**	**2013**	26	1	1	2	5	31
	**2014**	19	1	1	2	4	5
	**2015**	2	1	1	1	1	1
	**2016**	17	1	4	4	16	16
	**2017**	18	1	4	6	19	33
	**2018**	50	1	2	5	26	36
**Tetracyclines**	**2016**	1	26	26	26	26	26
	**2017**	3	1	1	5	5	5
	**2018**	10	1	1	1	5	24
**Trimethoprim**	**2013**	26	1	1	2	5	31
	**2014**	19	1	1	2	4	5
	**2015**	2	1	1	1	1	1
	**2016**	17	1	4	4	16	16
	**2017**	18	1	4	6	19	33
	**2018**	50	1	2	5	26	36

Supplement 1, Table 3: Major measures of the statistical distribution of the estimated weight in kg of the broiler chickens at the start of treatment per antimicrobial class

Antimicrobial class	Body weight in kg at start of treatment							
	number of records	mean	min	IQR	25% quantile	median	75% quantile	Max
**Aminoglycosides**	982	0.055	0.042	0.015	0.042	0.042	0.057	1.082
**Beta-Lactams**	845	0.894	0.042	1.254	0.160	0.929	1.414	2.429
**Fluoroquinolones**	136	0.231	0.042	0.077	0.057	0.073	0.134	2.334
**Lincosamides**	984	0.055	0.042	0.015	0.042	0.042	0.057	1.082
**Macrolides**	76	1.024	0.042	0.628	0.786	0.929	1.414	2.334
**Polypeptides**	1,087	0.887	0.042	1.390	0.111	0.856	1.501	2.524
**Sulfonamides**	132	0.376	0.042	0.252	0.042	0.091	0.294	2.144
**Tetracyclines**	14	0.296	0.042	0.069	0.042	0.077	0.111	1.244
**Trimethoprim**	132	0.376	0.042	0.252	0.042	0.091	0.294	2.144

Supplement 1, Table 4: Major measures of the statistical distribution of the estimated weight in kg of the broiler chickens at the start of treatment per antimicrobial class over the years

Antimicrobial class	year	Body weight in kg at start of treatment							
		number of records	mean	min	IQR	25% quantile	median	75% quantile	Max
**Aminoglycosides**	**2013**	94	0.058	0.042	-	0.042	0.042	0.042	1.082

Antimicrobial class	year	Body weight in kg at start of treatment							
		number of records	mean	min	IQR	25% quantile	median	75% quantile	Max
	**2014**	140	0.048	0.042	-	0.042	0.042	0.042	0.111
	**2015**	148	0.057	0.042	0.015	0.042	0.042	0.057	0.929
	**2016**	209	0.053	0.042	0.015	0.042	0.042	0.057	0.111
	**2017**	211	0.055	0.042	0.015	0.042	0.042	0.057	0.256
	**2018**	180	0.057	0.042	0.015	0.042	0.042	0.057	0.381
**Beta-Lactams**	**2013**	163	1.022	0.042	0.695	0.719	0.929	1.414	2.144
	**2014**	118	0.998	0.042	1.254	0.336	1.043	1.590	2.429
	**2015**	152	0.717	0.042	1.025	0.057	0.786	1.082	2.334
	**2016**	161	1.002	0.042	1.110	0.480	0.929	1.590	2.334
	**2017**	136	0.987	0.042	1.185	0.405	0.929	1.590	2.334
	**2018**	115	0.579	0.042	0.962	0.042	0.256	1.004	2.050
**Fluoroquinolones**	**2013**	52	0.189	0.042	0.069	0.042	0.073	0.111	1.863
	**2014**	14	0.103	0.042	0.103	0.057	0.091	0.160	0.189
	**2015**	19	0.507	0.042	1.202	0.042	0.091	1.244	2.334
	**2016**	17	0.194	0.042	0.034	0.057	0.073	0.091	1.956
	**2017**	22	0.094	0.042	0.018	0.073	0.091	0.091	0.220
	**2018**	12	0.425	0.042	0.618	0.057	0.126	0.676	1.863
**Lincosamides**	**2013**	94	0.058	0.042	-	0.042	0.042	0.042	1.082
	**2014**	140	0.048	0.042	-	0.042	0.042	0.042	0.111
	**2015**	148	0.057	0.042	0.015	0.042	0.042	0.057	0.929
	**2016**	209	0.053	0.042	0.015	0.042	0.042	0.057	0.111
	**2017**	211	0.055	0.042	0.015	0.042	0.042	0.057	0.256
	**2018**	182	0.057	0.042	0.015	0.042	0.050	0.057	0.381
**Macrolides**	**2013**	49	0.995	0.042	0.961	0.719	0.929	1.680	2.334
	**2014**	6	0.814	0.535	0.547	0.535	0.824	1.082	1.082
	**2015**	4	0.987	0.929	0.117	0.929	0.929	1.046	1.162
	**2016**	4	0.841	0.786	0.109	0.786	0.786	0.895	1.004
	**2017**	13	1.300	0.655	0.508	1.082	1.082	1.590	1.863
**Polypeptides**	**2013**	235	0.902	0.042	1.194	0.220	0.856	1.414	2.334
	**2014**	149	0.942	0.042	1.341	0.160	0.929	1.501	2.239
	**2015**	178	0.765	0.042	1.105	0.057	0.721	1.162	2.334
	**2016**	177	0.993	0.042	1.254	0.336	0.929	1.590	2.334
	**2017**	199	0.961	0.042	1.401	0.189	0.929	1.590	2.429
	**2018**	149	0.731	0.042	1.444	0.057	0.429	1.501	2.524
**Sulfonamides**	**2013**	26	0.154	0.042	0.069	0.042	0.050	0.111	1.680
	**2014**	19	0.069	0.042	0.049	0.042	0.057	0.091	0.111
	**2015**	2	0.042	0.042	-	0.042	0.042	0.042	0.042
	**2016**	17	0.290	0.042	0.444	0.091	0.091	0.535	0.535
	**2017**	18	0.513	0.042	0.628	0.091	0.134	0.719	1.863
	**2018**	50	0.601	0.042	1.187	0.057	0.113	1.244	2.144
**Tetracyclines**	**2016**	1	1.244	1.244	-	1.244	1.244	1.244	1.244
	**2017**	3	0.088	0.042	0.069	0.042	0.111	0.111	0.111

Antimicrobial class	year	Body weight in kg at start of treatment							
		number of records	mean	min	IQR	25% quantile	median	75% quantile	Max
	**2018**	10	0.264	0.042	0.069	0.042	0.042	0.111	1.082
**Trimethoprim**	**2013**	26	0.154	0.042	0.069	0.042	0.050	0.111	1.680
	**2014**	19	0.069	0.042	0.049	0.042	0.057	0.091	0.111
	**2015**	2	0.042	0.042	-	0.042	0.042	0.042	0.042
	**2016**	17	0.290	0.042	0.444	0.091	0.091	0.535	0.535
	**2017**	18	0.513	0.042	0.628	0.091	0.134	0.719	1.863
	**2018**	50	0.601	0.042	1.187	0.057	0.113	1.244	2.144
